# Impact of a physician-staffed helicopter on a regional trauma system: a prospective, controlled, observational study

**DOI:** 10.1111/aas.12052

**Published:** 2013-01-07

**Authors:** R Hesselfeldt, J Steinmetz, H Jans, M-L B Jacobsson, D L Andersen, K Buggeskov, M Kowalski, M Præst, L Øllgaard, P Höiby, L S Rasmussen

**Affiliations:** 1 Department of Anaesthesia Section 4231, Copenhagen University Hospital, RigshospitaletCopenhagen, Denmark; 2 Department of Emergency Medicine, Køge HospitalKøge, Denmark; 3 Department of Emergency Medicine, Hillerød HospitalHillerød, Denmark; 4 Department of Emergency Medicine, Slagelse HospitalSlagelse, Denmark; 5 Department of Emergency Medicine, Holbæk HospitalHolbæk, Denmark; 6 Department of Anaesthesia, Roskilde HospitalRoskilde, Denmark; 7 Department of Anaesthesia, Nykøbing Falster HospitalNykøbing Falster, Denmark; 8 Department of Emergency Medicine, Næstved HospitalNæstved, Denmark; 9 Department of Forensic Medicine, Section of Forensic Pathology, Copenhagen UniversityCopenhagen, Denmark; 10 Helicopter Emergency Medical ServiceRingsted, Denmark

## Abstract

**Introduction:**

This study aims to compare the trauma system before and after implementing a physician-staffed helicopter emergency medical service (PS-HEMS). Our hypothesis was that PS-HEMS would reduce time from injury to definitive care for severely injured patients.

**Methods:**

This was a prospective, controlled, observational study, involving seven local hospitals and one level I trauma centre using a before and after design. All patients treated by a trauma team within a 5-month period (1 December 2009–30 April 2010) prior to and a 12-month period (1 May 2010–30 April 2011) after implementing a PS-HEMS were included.

We compared time from dispatch of the first ground ambulance to arrival in the trauma centre for patients with Injury Severity Score (ISS) > 15. Secondary end points were the proportion of secondary transfers and 30-day mortality.

**Results:**

We included 1788 patients, of which 204 had an ISS > 15. The PS-HEMS transported 44 severely injured directly to the trauma centre resulting in a reduction of secondary transfers from 50% before to 34% after implementation (*P* = 0.04). Median delay for definitive care for severely injured patients was 218 min before and 90 min after implementation (*P* < 0.01). The 30-day mortality was reduced from 29% (16/56) before to 14% (21/147) after PS-HEMS (*P* = 0.02). Logistic regression showed PS-HEMS had an odds ratio (OR) for survival of 6.9 compared with ground transport.

**Conclusions:**

Implementation of a PS-HEMS was associated with significant reduction in time to the trauma centre for severely injured patients. We also observed significantly reduced proportions of secondary transfers and 30-day mortality.

Preventing trauma-related deaths remains a major challenge for the health care system.* Intermediate admission of severely injured trauma patients to a local hospital facility can cause delay in definitive care, and direct transport to a tertiary trauma centre (TC) is associated with improved outcome.^1^,^2^ Despite this, local emergency medical services (EMS) often bring severely injured patients to the nearest hospital.^3^,^4^

The Swiss-German physician-staffed helicopter emergency medical service (PS-HEMS) model was introduced and adapted in Scandinavia decades ago, as was the use of anaesthesiologists as pre-hospital emergency physicians.^5^ Trauma patients are thought to benefit from such advanced pre-hospital systems,^6^,^7^ and HEMS have been associated with a reduced mortality.^8^,^9^

Nevertheless, the current literature is often limited by retrospective study designs, and the heterogeneity of trauma systems complicate generalisation of conclusions. Moreover, the risk of helicopter accidents^10^,^11^ and increased cost^12^ of helicopter-based systems compared with conventional EMS request a thorough documentation of the effect. Hence, more data from Europe/Scandinavia are needed to illuminate the impact of PS-HEMS on time to definitive care, triage, and mortality for the regional trauma population.

We used the implementation of the first Danish PS-HEMS to conduct a prospective study, aiming to evaluate the pre-hospital trauma system in eastern Denmark, using a ‘before’ and ‘after’ design.

We hypothesised that the implementation of a PS-HEMS would reduce time from injury to definitive care at the trauma centre. In addition, we sought to assess whether the PS-HEMS would be associated with a reduced number of secondary transfers and reduced 30-day mortality for severely injured trauma patients.

## Methods

This was a prospective, controlled, observational, study, involving seven local emergency departments (non-trauma centres) and one regional level I equivalent trauma centre.

### Regional trauma system

The PS-HEMS operated in daylight hours (mean 11.3 h per day) in a flat rural area, covering 8400 km^2^ of eastern Denmark with a population of approximately 1.1 million, and a maximum driving distance to the trauma centre of 185 km. The dispatch centre used a designated protocol that stated that PS-HEMS should be primarily dispatched for (1) trauma with suspected severe injury (e.g. ejection from vehicle, high-speed MC accidents, and fall from > 4 meters), (2) trauma with reduced consciousness regardless of mechanism, (3) age under 2 years suffering trauma, (4) serious horseback riding accidents, and (5) mass casualty incidents. In addition, the expected driving distance to the trauma centre in Copenhagen for a ground unit should exceed 30 min. In addition, the protocol stated that the PS-HEMS could be dispatched secondarily, based on information from EMS providers on scene suspecting need for specialised care.

The existing regional EMS system consisted of ground units staffed with personnel on three competence levels. Level 1 is basic life support providers. Level 2 and 3 providers are all pre-hospital trauma life support certified with differentiated authority to administer intravenous fluid and medication. None has competence in tracheal intubation. Level 3 (paramedics) providers are allowed to insert laryngeal mask airway. The EMS units brought the trauma patients to the nearest hospital but were allowed to transport patients directly to the trauma centre after permission from a physician. Five mobile emergency care units (MECU) were available at various locations, staffed with anaesthesiologists on consultant level or anaesthetic nurses. Because of a regional political decision, four MECU units were omitted onwards from 1 March 2011 (last 2 months of our study).

The PS-HEMS was implemented on 1 May 2010. It was the first civilian HEMS in Denmark and was manned with an anaesthesiologist on consultant level, a flight paramedic, and a pilot. The seven local hospitals consisted of five level III equivalent and two level IV equivalent facilities, all having emergency departments and a protocol for trauma team activation.

### The study population

The study was conducted between 1 December 2009 and 30 April 2011, according to a pre-planned protocol.

We registered patients consecutively if treated by a trauma team in two periods: a control period of 5 months before PS-HEMS implementation (1 December 2009 until 30 April 2010) and a 12-month intervention period (1 May 2010 until 30 April 2011) after implementation. We included all trauma patients and excluded patients who were transported to the emergency department (ED) by private means or were brought in by the police. We also excluded those who upon arrival in the ED were categorised as non-trauma patients. Patients with burns were not included.

A regional trauma registry did not exist at the time of the study, though we gathered 24 of the 36 core data variables as outlined in the Utstein Template.^13^ In all of the eight centres, a local investigator was appointed, and the emergency department staff was informed and instructed to fill out a designated study registration sheet on all trauma team calls and gather copies of the ambulance records. Collected data included transport mode, demographics, time intervals (from EMS activation until destination hospital), mechanism of injury, type of injury, highest level of triaging authority on scene, and initial on-scene and in-hospital vital signs. Furthermore, we collected ambulance reports, hospital records, and autopsy reports. One person (R. H.) centrally calculated the Injury Severity Score (ISS)^14^ and the New ISS (NISS)^15^ after having rated all injuries according to the Abbreviated Injury Scale (AIS 2005© update 2008). To address potential differences in risk factors associated with poor outcome between the two groups, we used the Trauma ISS (TRISS).^16^ Based on a baseline population from the Major Trauma Outcome Study (MTOS),^17^ the TRISS integrates weighted data on age, ISS, and physiological status [Revised Trauma Score, (RTS)] into a probability of survival. To assess the ‘system performance’ in each group, three analyses are reported (W, Z, and M statistics). W quantifies the difference between the actual and predicted survival of patients and provides a number of unexpected survivors or unexpected deaths per 100 patients. The Z statistic tests whether W is significantly different from 0. A Z < –1.96 is defined as significantly lower and Z > 1.96 as significantly higher observed mortality than expected. By matching the distribution of the included patients’ probability of survival in each group, with the distribution in MTOS, the M statistic supports/rejects case mix similarity and by that the usage of TRISS. M > 0.88 defined good correlation. We used the first recorded vital signs on scene in calculating the RTS.^16^,^18^ W and Z are reported on patients with ISS > 15 for whom missing RTS values were replaced with ‘normal values’ [systolic blood pressure (SBP) 120, respiratory rate (RR) 12, Glasgow Coma Scale (GCS) 15].^20^

### Outcome measures

The primary end point was the time from the dispatch of the first ground EMS to the arrival in the TC trauma bay, for patients with severe injury (defined as an ISS > 15) arriving within 48 h from injury in the trauma centre.

Secondary end points were the proportion of severely injured patients secondarily transferred to the trauma centre, 30-day mortality, and on-scene triage. On-scene under-triage was defined as patients with ISS > 15 transported to the local hospital, and over-triage as patients with ISS < 15 transported directly to the trauma centre.

### Statistical analysis

Continuous data are reported as medians with 5–95% percentiles and compared using the Mann–Whitney test.

Categorical data are reported as numbers (%) and 95% confidence interval (CI) for mortality rates. Groups were compared using χ^2^ test or Fisher's exact test where appropriate.

A logistic regression analysis was conducted to assess survival chance after 30 days. This included transport mode (PS-HEMS vs. ambulance), age, and ISS. Odds ratios (ORs) were reported with 95% CI. Patients examined on scene by the PS-HEMS physician but transported by ground ambulance were not included in the logistic regression analysis.

We considered that a clinically relevant difference in time from dispatch of the first ground EMS to arrival in the TC trauma bay would be 30 min, and we estimated that the standard deviation would be 36 min, derived from a previous regional study.^4^ We estimated that 200 patients would be enrolled in the primary end point analysis. Thus, a 30-min difference could be detected with a power of > 95% at the 5% significance level accordingly.

Data were analysed using SAS version 9.1 (SAS institute, Inc., Cary, NC, USA). *P* < 0.05 was considered statistically significant.

Prior to the study, we received approval from the Danish Data Protection Agency (j. nr: 2009-41-4122) and the National Board of Health (j. nr: 7–604-04-2/128/HKR). According to Danish law, approval from the Ethics Committee and collection of informed consent were not required for this study.

## Results

**Fig. 1:**
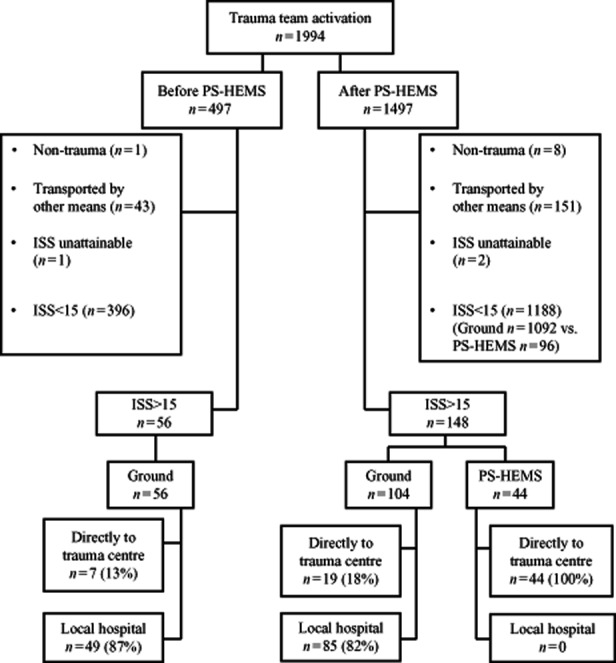
Flow chart of included trauma patients and distribution of severely injured [Injury Severity Score (ISS) > 15] between ground and physician-staffed helicopter emergency medical service (PS-HEMS) transport.

In the study period, trauma teams were activated for 1994 patients, of whom we included 1788 (Fig. 1). Of these, 204 were severely injured with an ISS > 15, 56 patients in the 5-month period before PS-HEMS implementation, and 148 in the 12-month period after implementation.

The severely injured patients were significantly older in the before implementation group, but no significant differences in gender, ISS, NISS, severe head trauma (head AIS > 3), trauma type, trauma mechanism, or proportion of pre-hospital intubation were found (Table 1).

**Table 1:** Characteristics for severely injured patients (Injury Severity Score (ISS) > 15)

	Before PS-HEMS implementation (*n* = 56)	After PS-HEMS implementation (*n* = 148)	*P* value
Age (years) (5–95% range)	56 (21–88)	47 (15–81)	0.04
Male gender	39 (70%)	104 (70%)	0.93
ISS (5–95% range)	25 (17–45)	25 (16–43)	0.18
NISS (5–95% range)	33 (17–50)	29 (17–57)	0.42
Head AIS > 3	23 (41.1%)	47 (31.8%)	0.21
Type			
Blunt	51 (91%)	142 (96%)	0.17
Penetrating	5 (9%)	6 (4%)	
Mechanism			
Road traffic accident	30 (53%)	83 (56%)	0.78
Fall > 2 meters	10 (18%)	28 (19%)	
Fall < 2 meters	7 (12%)	10 (7%)	
Assault	2 (4%)	7 (5%)	
Sports	1 (2%)	1 (1%)	
Other	6 (11%)	19 (13%)	
Triage authority on scene†			
Unknown	2 (4%)	2 (2%)	
EMS	22 (39%)	59 (40%)	
MECU-nurse	2 (4%)	3 (2%)	
MECU-physician	30 (53%)	39 (26%)	
PS-HEMS	NA	45 (30%)‡	
Pre-hospital endotracheal intubation	8 (14.3%)	34 (23.0%)	0.17
30-day mortality	16 (29%) (18–42%, 95%CI)	21 (14%)§ (9–21%, 95%CI)	0.02
30-day mortality (daytime: 08:00–20:00 hours)	14/42 (33.3%) (21–49%, 95%CI)	16/98 (16.3%) (10–25%, 95%CI)	0.02
Overall 30-day mortality¶ *n* = 1766	18/448 (4.0%) (3–6%, 95%CI)	29/1318 (2.2%) (2–3%, 95%CI)	0.04

† Only the upper authority registered if more than one unit present. PS-HEMS physician was registered as triaging authority, if both PS-HEMS and MECU were present.

‡ One patient was triaged but not transported by PS-HEMS.

§ *n* = 147. One patient was lost to follow-up.

¶ Regardless of ISS.

PS-HEMS, physician-staffed helicopter emergency medical service; ISS, Injury Severity Score; NISS, New Injury Severity Score; AIS, Abbreviated Injury Scale; EMS, emergency medical services; MECU, mobile emergency care unit; CI, confidence interval; NA, not applicable.

**Fig. 2:**
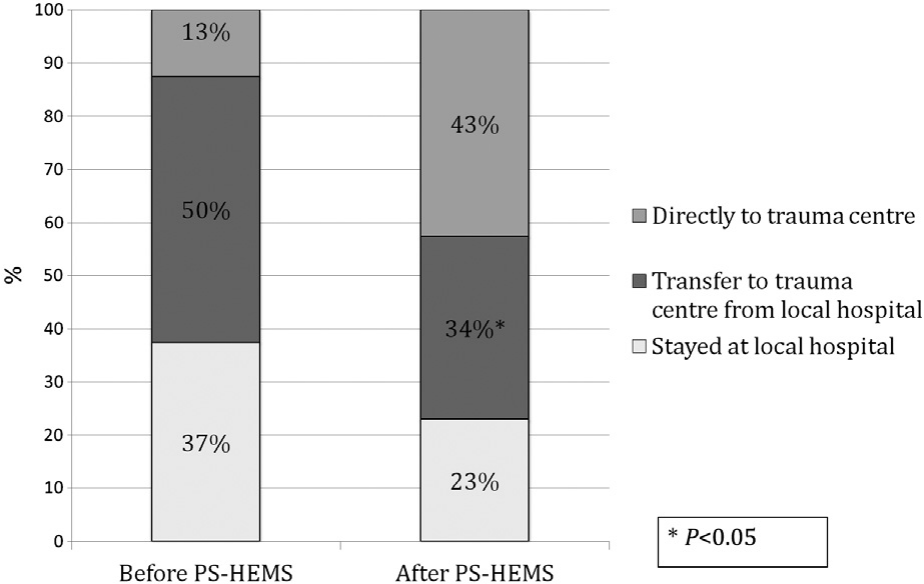
Triage of severely injured patients (Injury Severity Score (ISS) > 15) to hospital facility before and after implementation of a physician-staffed helicopter emergency medical service (PS-HEMS). *P < 0.05.

The median time from first EMS dispatch to arrival in the trauma centre was 218 min and 90 min before and after implementation, respectively (*P* < 0.01) (Table 2). After the PS-HEMS started operating, the proportion of secondary transfers of severely injured to the TC dropped from 50% to 34% (*P* = 0.04) (Fig. 2).

**Table 2:** Time intervals in minutes (5–95% range) for severely injured patients (Injury Severity Score > 15)

	Before PS-HEMS (*n* = 56)	After PS-HEMS (*n* = 148)	*P* value
Time from emergency medical system dispatch to arrival at initial hospital	52 (21–103) (*n* = 51)	60 (24–96) (*n* = 144)	0.03
Time from emergency medical system dispatch to arrival in trauma centre (< 48 h)	218 (54–832) (*n* = 29)	90 (57–458) (*n* = 107)	< 0.01
Time from emergency medical system dispatch to arrival in trauma centre (< 48 h) GRD only.	218 (54–832) (*n* = 29)	219 (59–925) (*n* = 63)	0.63

Time intervals in minutes (5–95% range) for all patients received by trauma team			

	Before PS-HEMS (*n* = 497)	After PS-HEMS (*n* = 1497)	
Time from emergency medical system dispatch to arrival at initial hospital	53 (29–88) (*n* = 426)	53 (28–89 (*n* = 1252)	0.97
Time from emergency medical system dispatch to arrival in trauma centre (< 48 h)	185 (66–735) (*n* = 42)	78 (53–322) (*n* = 249)	< 0.01

Time intervals in minutes (5–95% range) for severely injured patients (Injury Severity Score > 15)			

	Ground (*n* = 160)	PS-HEMS (*n* = 44)	
Time from emergency medical system dispatch to arrival at initial hospital	50 (22–98) (*n* = 151)	76 (56–96) (*n* = 44)	< 0.01
Time from emergency medical system dispatch to arrival in trauma centre (< 48 h)	219 (57–925) (*n* = 92)	76 (56–96) (*n* = 44)	< 0.01
Time from emergency medical system dispatch to arrival in trauma centre (when initial hospital)	82 (52–140) (*n* = 26)	76 (56–96) (*n* = 44)	0.65

PS-HEMS, physician-staffed helicopter emergency medical service; GRD, ground ambulance.

The 30-day mortality of the severely injured was significantly reduced from 29% before PS-HEMS implementation to 14% in the year after implementation (*P* = 0.02). Accordingly, the overall mortality, regardless of ISS, was also significantly lower in the after period (Table 1).

Compared with all ground patients in the 17-month period, PS-HEMS patients were more severely injured (ISS 9 vs. 1, *P* < 0.01), but no significant difference was found in 30-day mortality [5/141 (3.6%) vs. 42/1625 (2.6%) *P* = 0.50]. The same pattern was seen in patients with ISS > 15 (median ISS 26 vs. 25, *P* = 0.049, in terms of 30-day mortality 9.1% vs. 20.8%, *P* = 0.08, respectively).

There were 1.4% (25/1788) patients lost to follow-up, but only one was severely injured.

During daytime (08:00–20:00 h) in the 12-month after period, there were 99 severely injured. Of those, 42 were (42%) triaged by PS-HEMS.

During the 17-month observation period, severely injured patients were under-triaged to the local hospital in 79/81 (97.5%) by conventional ground ambulance, 2/5 (40%) by non-physician-staffed MECU, 49/69 (71%) by physician-staffed MECU, and 1/45 (2.2%) by PS-HEMS.

The logistic regression analysis including all trauma patients with complete set of data (*n* = 1726) in the 17-month period revealed that treatment and transport with PS-HEMS was associated with a significantly higher chance of survival compared with transport by ground ambulance (OR = 6.9, 95% CI 1.48-32.5 *P* = 0.01). By replacing ISS with NISS in the logistic regression analysis, the results were (OR = 5.1, 95% CI 1.3–19.7), and by adding head AIS > 3 as explanatory variable together with NISS, the PS-HEMS still increased the chance of survival significantly (OR = 4.9, 95% CI 1.3–19.3).

For performing the TRISS analysis, we found an acceptable distribution of severity mix in the before and after group (M = 0.94 vs. M = 0.93). The actual mortality was significantly lower than predicted after implementation of the helicopter service (Z = 1.24 vs. Z = –2.58). According to the W statistic, there were 6.4 unexpected survivors per 100 patients in the period after PS-HEMS implementation compared with the baseline MTOS population. Missing physiological values were inserted in 23% (13/56) vs. 14% (21/148) of the cases in the before and after group for the calculation of TRISS.

## Discussion

Time from injury to definitive care at the trauma centre was significantly reduced for severely injured trauma patients after implementation of a PS-HEMS. The proportion of secondary transfers decreased significantly from 50% to 34%. In addition, the 30-day mortality for the regional group of severely injured trauma patients was reduced from 28.6% to 14.3% in the before and after PS-HEMS groups, respectively.

The prospective and regional population-based design strengthens this study and allows assessment of the important group of patients staying at the local hospitals.^21^ By using a before and after design, we could ‘control’ the impact of PS-HEMS with a recent historic population, instead of performing a traditional ground vs. air study. Moreover, we used actual EMS transport times > 30 min in inclusion of ground patients, thereby assessing only those patients who were eligible for helicopter transport, i.e. urban trauma patients were not included.

Every citizen in Denmark is provided with a unique central-registered personal identification number at birth.^22^ This number ensures highly valid follow-up compared with the large-scale North American registry studies.^8^

A number of limitations must be taken into account when interpreting the results of this study. In a non-randomised study, there is an inherent risk of selection bias. A control period of five winter and spring months vs. a full year intervention period provides a risk of seasonal differences in the trauma population. However, we did not find any difference in the time to definitive care for ground transportation between the two periods. Accordingly, we have not been able to find studies documenting that injuries occurring during winter and spring have higher mortality.^23^,^24^

Changes in regional organisation or treatment performance according to time period could have been a confounder in this design. To our knowledge, the omission of the physician- and nurse-staffed MECU units 2 months before completion of the study was the only change in organisation.

Although the use of TRISS is recommended in HEMS trauma outcome studies,^25^ limitations to the analysis must be emphasised. When patients are intubated prior to hospital arrival, a valid in-hospital GCS scoring for the RTS is impossible. A solution to overcome this problem is the use of pre-hospital values before patients were intubated.^26^ Different alternatives have been used for substituting the missing values. We inserted normal values, which is the most conservative solution, as this will result in a higher probability of survival, and thereby underestimate the actual system performance. Even though we found 6.4 unexpected survivors per 100 patients in the ‘after’ period, this is an arbitrary quantification of performance because it is derived from a historic North American control group (the MTOS patients^17^). Although adjustments to the TRISS method have been proposed,^27^ no consensus of ‘golden standard’ exists. We therefore emphasise to interpret it with caution, though we do believe that the results from the TRISS analysis support the overall findings of improved survival after PS-HEMS implementation and reduce the chance that they are due to selection bias.

In addition to this, the logistic regression support that the improved survival is due to implementation of the PS-HEMS. However, the logistic regression is limited by the relatively few number of adjusting factors (age, ISS). ISS has been challenged by the less commonly used NISS, which has been found to be a better mortality predictor.^28^ Though ORs were still in favour of PS-HEMS transport when adjusting for NISS and severe head injuries (head AIS > 3).

We did not report or adjust for pre-injury co-morbidity status, which is a known risk factor^29^ and raises a chance of selection bias influencing the mortality analysis. Furthermore, we did not report disposition status and cannot rule out that the improved survival outcome after PS-HEMS implementation to be at the expense of functional deficits.

Finally, there is a risk of incomplete registration at the local hospitals. This potential information bias was diminished by several visits to the EDs during the entire study period and by having one or more dedicated persons at every site screening for trauma patients and prospectively checking data completeness.

One of the key findings in our study was the profound under-triage of the severely injured by all types of ground EMS units, preferably transporting patients to the local hospital instead of the trauma centre. Even though protocols determine that suspected severely injured patients should be transported directly to the trauma centre, there are barriers to fulfil this. A recent study showing that transfer from a non-trauma centre is associated with a considerable delay in transport to the trauma centre also found pathological patient characteristics on scene indicating a need for direct level I care.^2^ This suggests that other factors influence the EMS choice of receiving facility.

One could argue that the reduction in field under-triage found in our study was due to bringing a physician to the scene in the PS-HEMS period, but the actual percentage of injuries with a physician as triaging authority was the same in the two periods. A possible explanation could be that PS-HEMS personnel are more experienced, trained, and confident in triaging, treating, and transporting severely injured patients over distance. A Norwegian study by Rehn et al.^30^ showed higher ability to recognise severe trauma by anaesthetist-staffed MECU/HEMS but did not differentiate between ground and air units, and did not include under-triage to local hospital. The PS-HEMS base in our study was over 65 kilometres from the trauma centre, so it was not just a result of bringing the patient ‘return to base’.

Another explanation for the observed field under-triage by ground EMS in our study could be that unstable patients are brought to the nearest local hospital for stabilisation. However, the time spent at the local hospital is often used for non-therapeutic examinations, which are costly and delay time to definitive care.^31^–^33^

A previous study, from our region, found that approximately 40% of the severely injured patients outside the urban area close to the trauma centre were secondarily transferred to the TC, and the median time delay from arrival at the local hospital until arrival at the TC was 198 min.^4^ These findings are consistent with our results in the 5-month pre-PS-HEMS period and indicate that this was representative. The clinical impact of field under-triage was illustrated in a European study, where they observed a doubled time from EMS activation to the start of emergency neurosurgical interventions on secondarily transferred trauma patients compared with direct TC transport, proposing a need for better pre-hospital triage.^34^

We did not observe under-triage by the PS-HEMS, but a considerable over-triage was present. Only 31% of the PS-HEMS transported patients had an ISS > 15. Although this over-triage is consistent with data from other HEMS,^35^,^36^ these figures indicate an overutilisation and the potential of a more efficient use.

HEMS has been found to be associated with significantly shorter transport and total pre-hospital time than ground transportation.^35^ Previous findings in a prospective regional Italian study support the results in our study. They compared time to definitive care and mortality for severely injured patients transported by either a non-urban, daylight-operating physician-staffed helicopter or ground ambulance, and found higher 30-day mortality for patients transported by ground (38% vs. 12%). They also found that total times to the trauma centre were shorter (55 min vs. 162 min) when transported directly by air.^37^

Other studies have reported longer accident to hospital time for HEMS.^9^,^38^

Most HEMS research focus on mortality, and registry studies have found an association between HEMS transport and lower mortality,^8^,^9^,^20^,^39^ but the causality is uncertain.

Clearly, the time gain in our study was due to the direct transport of PS-HEMS patients to the trauma centre. As the relationship between direct transfer to level I care and mortality seems to be well documented, this might be the main explanation of our findings.^38^,^40^,^41^

Data presented in this study support the use of a regional PS-HEMS and make a valuable contribution to the debate for EMS administrators, politicians, and policy makers. We suggest that future research should focus on minimising EMS field under-triage, long-term morbidity for HEMS vs. ground, and optimising HEMS dispatch criteria in order to identify patients needing level I trauma care.

## Conclusions

In conclusion, implementation of a physician-staffed helicopter was associated with significantly reduced delay for arrival at the level I trauma centre of severely injured trauma patients. The proportion of secondary transfer and 30-day mortality were also significantly reduced.

## Footnotes

* Sethi D, Racioppi F, Baumgar en I, Vida P. Injuries and violence in Europe: why they matter and what can be done. WHO Regional Office Europe; 2006. http://www.euro.who.int/__data/assets/pdf_file/0005/98762/E88037.pdf [Accessed 19 December 2012].
